# Intraoperative Assessment of High-Risk Thyroid Nodules Based on Electrical Impedance Measurements: A Feasibility Study

**DOI:** 10.3390/diagnostics12122950

**Published:** 2022-11-25

**Authors:** Jalil Beheshti Firoozabadi, Reihane Mahdavi, Khosro Shamsi, Hossein Ataee, Abdollah Shafiee, Hojat Ebrahiminik, Hossein Chegini, Parisa Hoseinpour, Afshin Moradi, Narges Yousefpour, Faeze Aghaei, Ali Fardoost, Alireza Ghelichli, Hadi Mokhtari Dowlatabad, Farzane Hajighasemi, Nafiseh Sami, Seyed Rouhollah Miri, Mohammad Esmaeil Akbari, Mohammad Abdolahad

**Affiliations:** 1 Cancer Research Center, Shahid Beheshti University of Medical Sciences, Tehran 14166-34793, Iran; 2 Nano Bioelectronics Devices Laboratory, School of Electrical and Computer Engineering, Faculty of Engineering, University of Tehran, Tehran 14399-57131, Iran; 3 Cancer Electronics Research Center, University of Tehran and Tehran University of Medical Sciences Imam Khomeini Hospital, Tehran 14197-33141, Iran; 4 Department of Electrical Engineering, Amirkabir University of Technology, Tehran 15916-34311, Iran; 5 Department of Surgery, Farmanieh Hospital, Tehran 19537-34411, Iran; 6 Department of Internentional Radiology and Radiation Sciences Research Center, Aja University of Medical Sciences, Tehran 14117-18541, Iran; 7 Interventional Radiology Department, Tirad Imaging Institute, Tehran 15867-36513, Iran; 8 Department of Pathology, Breast Cancer Research Center, Motamed Cancer Institute, ACECR, Tehran 15179-64311, Iran; 9 Department of Pathology, Shohada Hospital, Shahid Beheshti University of Medical Sciences, Tehran 14166-34793, Iran; 10 Department of Medicine, Islamic Azad University of Medical Sciences, Tehran 19395-1495, Iran; 11 Department of Surgical Oncology, Tehran University of Medical Science, Tehran 14176-14411, Iran; 12 Cancer Institute, Imam-Khomeini Hospital, Tehran University of Medical Sciences, Tehran 14166-34793, Iran

**Keywords:** electrical impedance spectroscopy, fine needle aspiration, thyroid nodule, atypia of undetermined significance (AUS), impedance phase slope (IPS), surgery

## Abstract

Precise diagnosis of thyroid nodules is challenging due to non-diagnostic/inconclusive results and uncertainties about the malignancy of follicular neoplasms (FNs), even in frozen-section pathology. Therefore, surgical management, especially in Bethesda III and IV categories, may be complicated, and sometimes a second surgery may be required. The Thyroid Nodule Impedance Measurement System (TN-IMS) consists of a metallic patch attached to submental skin and a G20 I.V. cannula inserted into the targeted nodules. Two impedance-based parameters named Z_1kHz_ and impedance phase slope (IPS) in 100 kHz to 500 kHz of the thyroid nodules are recorded and compared with their histopathological results as the gold standard. TN-IMS was intra-surgically applied to 103 human thyroid nodules and normal thyroid tissues. A remarkable consistency between defined co-ranges of Z_1kHz_/IPS and the histopathological status of specimens was achieved (*p* < 0.001). Based on these measurements, it was concluded that intraoperative bioelectrical impedance scanning of thyroid nodules would be a helpful complementary approach to detecting high-risk excision-required thyroid nodules.

## 1. Introduction

Although thyroid cancer currently ranks as the ninth most prevalent cancer in the world, its incidence has increased dramatically in the last 20 years, and this increase has been faster than any other cancer [1,2,3]. such evolution may be primarily in correlation with improvements in the ability to detect small, clinically occult papillary cancers [4,5].

While many improvements in the early diagnosis of thyroid cancers (sequential sonography, FNA, and nuclear scans) have positively impacted the detection of high-risk thyroid disease, some of these lesions are still challenging in pathological diagnostics and induce drastic confusion in the surgeons’ decision about the required interventional procedure (follow-up, lobectomy, or total thyroidectomy).

Complications of thyroidectomy include unilateral or bilateral recurrent laryngeal nerve injury that leads to hoarseness and tracheotomy [6], temporary or permanent damage to the parathyroid that may lead to hypocalcemia requiring replacement of Calcium and vitamin D supplements, and bleeding after surgery, which may need re-exploration of the neck [7,8]. There are some solutions that have been reported in the literature to overcome these complications, such as loupes magnification and microsurgical techniques [9].

The gold standard for thyroid nodules diagnosis is ultrasound-guided fine-needle aspiration (FNA) [10,11]. A diagnostic sensitivity of 83–98% and specificity of 70–92% have been reported in the literature for this process [10], which is highly dependent on the skill of both the US operators and the cytologists [12]. However, the high rate of non-diagnostic (inadequate cells or hemorrhagic samples) and indeterminate cytology results of FNA samples remains critical [10,13]. Atypia of undetermined significance (AUS) and follicular lesion of undetermined significance (FLUS) occur in approximately 10–33.6% and 15–42% of all FNA samples, respectively [10]. However, in many cases, repeating FNA may result in uncertainty leading to unnecessary surgeries [14,15] or misdiagnosis, and does not seem to be a satisfactory solution [16]. 

Another critical issue in thyroid cancer detection are follicular or Hurthle cell lesions that are challenging and difficult to diagnose in FNA cytopathology, or even frozen-section histopathology of dissected mass, since only histopathological evidence of capsular and vascular invasion can confirm malignancy in such neoplasms [17,18,19]. Therefore, thyroid nodules scoring in the range of Bethesda III–IV may not only challenge radiologists and pathologists before surgery but also cause doubt for surgeons during surgery [20].

In recent years, core needle biopsy (CNB) from thyroid nodules has been recommended for previous inconclusive FNAs [21]. Also, some researchers have confirmed that CNB could overcome the limitations of FNA in these lesions [10,13,22,23]. However, the role of CNB has not been well established for the assessment of thyroid nodules with suspicious US features [10].

We designed and fabricated an electrical impedance-based system and fully calibrated it with histopathological results obtained in-vivo from 103 thyroid nodules or normal thyroid tissues in a cohort study of 55 patients. The so-called system, Thyroid Nodule Scanning (TN-IMS), is a precise, real-time, and low-invasive tool that can detect excision-required thyroid nodules during surgery to overcome the uncertainty of FNA. Further, we know that a probable frozen-section pathology could be undertaken on dissected nodules with no impact on pre-excisional diagnoses. 

This system records and analyses two electrical impedance features of Z_1kHz_ and IPS of the nodules by electrical impedance spectroscopy (EIS) between a metallic lead attached to the skin of the submental region and a G20 medical-grade needle inserted into the targeted nodule. It is well known that the dielectric properties of a biological specimen in response to alternating voltage are related to its composition, structure, health status, and physiological or pathological properties [24,25,26]. We investigate the probable difference in these two parameters between malignant/high-risk thyroid nodules recommended for excision (such as PTC, follicular carcinoma, and Hurthle cell carcinoma) and low-risk follow-up required nodules (such as Hashimoto thyroiditis, follicular adenoma, and goiters).

## 2. Materials and Methods

### 2.1. TN-IMS Measurement System and Mechanism

TN-IMS is a precision impedance-based measurement system that calculates and analyses impedance magnitude and phase diagrams of the tested media in a two-electrode configuration. First, a 0.4 V alternating voltage signal is applied to the tissue utilizing a two-electrode probe that stimulates the tissue to produce a current response signal recorded by the system. Then the impedance magnitude is calculated by dividing the voltage by current signal amplitudes. The impedance phase is defined according to the electrical current phase shift against the voltage signal. Two parameters of impedance magnitude in the frequency range of 1 kHz (Z_1kHz_) and impedance phase slope in the frequency ranges of 100 kHz to 500 kHz (IPS) are then extracted as two impedance-based features identifying intracellular, extracellular, and membranous abnormalities due to cancer developments. 

The stimulating/sensing probe has two different metallic electrodes to conduct electrical current through the body. One electrode is a G20 disposable I.V. cannula inserted in the suspicious target nodule. The other metallic electrode is a disposable ECG chest electrode with a pre-gelled Ag/AgCl sensor attached to the submental skin of the patient. The measurements take place in about 3 s, and the results are announced in real-time according to a predefined calibration according to histopathological examinations. A multiphysics simulation has been performed to evaluate several sensor sensitivity parameters reported in the supplementary document.

### 2.2. TN-IMS Measurement Protocol

**Figure 1:**
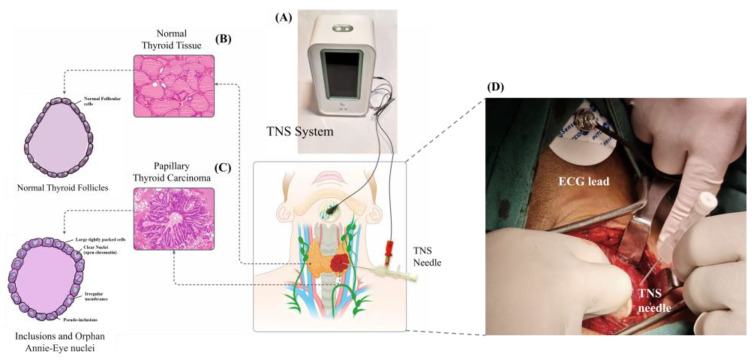
Protocols of TN-IMS measurement. (**A**) The TN-IMS system is in contact with a suspected thyroid nodule via two electrodes, a needle probe inside the nodule, and an ECG chest lead connected to the submental region. (**B**) The pathological structure of normal thyroid tissue is illustrated through an H&E assay and a schematic picture. (**C**) Pathological structure of a cancerous thyroid nodule presented by an H&E assay [27]. Inclusions and Orphan Annie-eye nuclei patterns are illustrated in a schematic. (**D**) The intraoperative application of TN-IMS needle probe.

After positioning the patient and making long-acting anesthesia, an incision is made, the skin flaps are raised, the strap muscles are mobilized, and the thyroid nodule is exposed by retractors. Then the sterilized ECG lead electrode is attached to the submental skin. The surgeon inserts the needle electrode into the suspicious nodule before ligating thyroid pole vessels to avoid any blood circulation change in the thyroid nodule (Figure 1).

Since TN-IMS (and any intraoperative device) has no decisive results, we used an individual disposable G20 cannula for each patient’s nodule regardless of its presurgical pathological evaluations to prevent seeding effects from malignant lesions.

The TN-IMS tested at least three points of each nodule with a measurement time of 3 s to cover all the nodule areas because of the heterogeneous nature of high-risk thyroid nodules [28]. The ECG lead was connected to the submental region at the beginning of surgery during preparation of the patient. The three measurements with TN-IMS and response extraction take less than one minute on each nodule, which is acceptable in surgery. Electrical measurements and analysis were then made and announced to the surgeon as a complementary device for surgical management. Each tested nodule is then sampled for precise evaluations after thyroid dissection. For multinodular thyroid glands, measurements may be done for each palpable nodule according to the surgeons’ assessment. The parameters extracted from each tested nodule were then compared with the histopathological diagnostics samples.

### 2.3. Statistical Analysis

To assess the significance of the differences between the IPS value and histopathological status of tumors, asymptotic significance, or *p*-value, of the chi-square, with a confidence interval of 95%, determining the statistical significance of the relationship was calculated. Receiver operating characteristic curve analysis was used to calculate the area under the curve, and a cut-off value for IPS was extracted. All statistical analyses were performed using commercially available software (IBM SPSS Statistics for Windows version 25).

### 2.4. Ethics

The project was performed according to the World Medical Association Ethics, Declaration of Helsinki, and the ethical principles and national standards for conducting Medical Research in Iran. All of the human tests were performed under the license of the Ethics Committee of Tehran University of Medical Sciences with the informed consent of candidate patients. Institutional review board (IRB) or research ethics committee (REC) approval and clinical trial registration numbers of the project are IR.TUMS.VCR.REC.1397.355, and IRCT20190904044697N7, respectively.

## 3. Results

**Figure 2:**
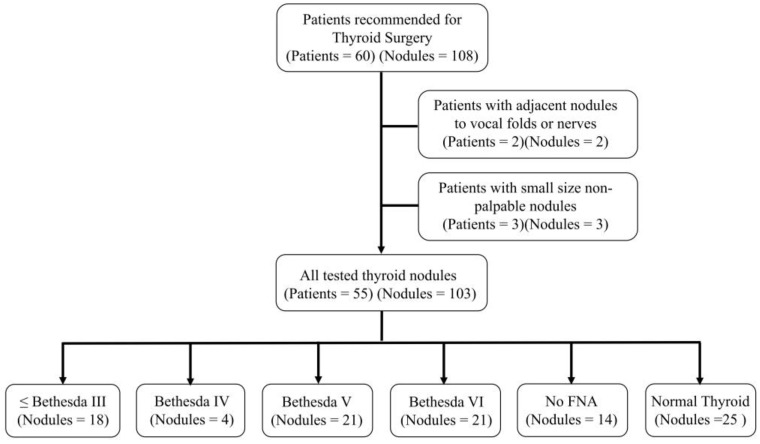
The study flow diagram shows patient exclusion.

Patient candidates for thyroid surgery were recruited in the study (*n* = 60). Due to conflicts about inserting the G20 I.V. Cannula in nodules adjacent to nerves or vocal folds and possible post-surgical complications, two patients were excluded from the study. Also, patients with small (<5 mm) nodules that are non-palpable by the surgeon during surgery were excluded because of uncertainty about the accurate insertion of the needle test in the nodule (*n* = 3). Therefore, the cohort study included 55 patients with 78 thyroid nodules of Bethesda III or lower (*n* = 18), Bethesda IV (*n* = 4), Bethesda V (*n* = 21), and Bethesda VI (*n* = 21) (Figure 2). Also, some nodules that were not detectable in sonographic evaluations but detected intraoperatively or unremarkable nodules in multinodular thyroids were tested intraoperatively during the study (*n* = 14). Some normal thyroid tissues (*n* = 25) were also tested by TN-IMS.

Medical documents of the research population confirm that 47 (85%) of the patients were female, while eight others (15%) were male (Table 1). The mean age for the cohort study and average nodule size are 43.3 and 18.9 mm, respectively. The current population was classified as Bethesda I (0.97%), II (7.77%), III (8.73%), IV (3.88%), V (20.38%), VI (20.38%) in cytopathology. The remaining 13.59% were nodules with no performed FNA, and 24.3% were normal thyroid tissues. The post-surgical histopathologic evaluations reported 60 (58.2%) malignant Papillary Thyroid Carcinoma (PTC) or micro-PTC nodules, 3 (2.9%) Hurthle Cell carcinoma (HCC), 1 (0.97%) Hurthle cell neoplasm (HCN), 14 (13.6%) low-risk benign nodules (such as Hashimoto thyroiditis, colloid goiter, etc.), and 25 (24.3%) normal thyroid tissue (Table 1).

The TN-IMS response was calculated by analyzing the dielectric properties of thyroid samples obtained via two impedimetric classification parameters of Z_1kHz_ and IPS. These two parameters have been used in previous studies on cancerous breast tumor margins and involved lymph nodes with promising results [28,29]. According to the surgeon’s diagnosis, these two parameters were recorded for each palpable thyroid nodule or any area that seemed suspicious. After preparing permanent pathology results of samples and nodules (as the gold standard), they were labeled positive and negative for cancer or a suspicious neoplasm lesion. 

**Figure 3:**
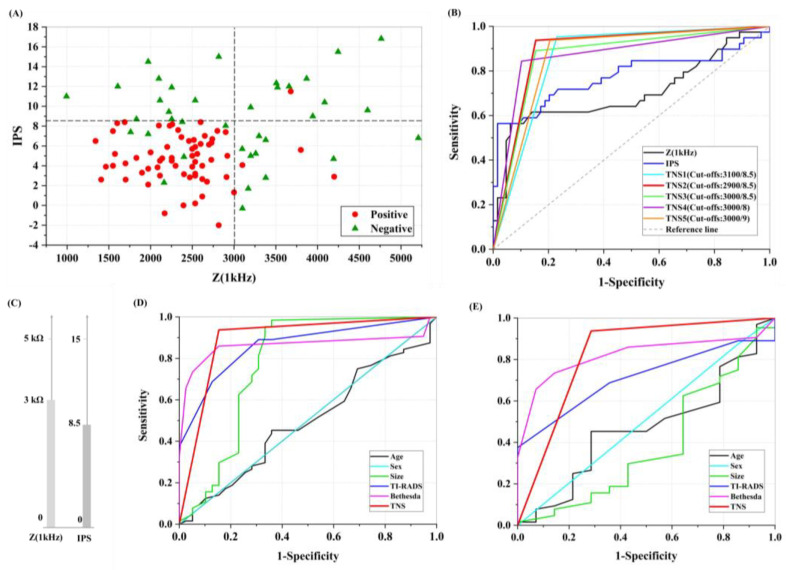
TN-IMS calibration and scoring. (**A**) A two-dimensional diagram representing Z_1kHz_ on X-axis and IPS on Y-axis for all tested samples defines a primary calibration cut-off set. The patterned rectangle illustrates the positive region with the most malignancy probability in thyroid samples. (**B**) The classification criteria for positive thyroid nodules. (**C**) The effect of changing calibration features cut-offs in AUC, sensitivity, and specificity. Most AUC and sensitivity/specificity compositions belong to the primarily defined calibration cut-offs. (**D**) Comparison of AUC, sensitivity, and specificity of clinical indications such as age, sex, nodule size, TI-RADS, Bethesda, and TN-IMS scores in all tested samples (including thyroid nodules and normal thyroid tissues). (**E**) AUC, sensitivity, and specificity of clinical indications such as age, sex, nodule size, TI-RADS, Bethesda, and TN-IMS score in only thyroid nodules.

For detecting the cut-off values of Z_1kHz_ and IPS, all the thyroid nodules and samples tested by TN-IMS were collected in a two-dimensional calibration graph (Z_1kHz_ on the X-axis and IPS on the Y-axis). Each thyroid nodule is represented by a point in the diagram and has a specific Z_1kHz_ and IPS. Also, each thyroid nodule has a permanent pathology diagnosis. All pathologically negative nodules are represented in green, and the red colored symbols declare positive nodules. Therefore, the primary cut-offs for malignant thyroid nodules can be estimated by observing the region where the red points are most abundant. It can be deduced from Figure 3A that almost all positive samples (red dots in the diagram) are confined to a rectangle with Z_1kHz_ and IPS cut-off values of 3000 ohms and 8.5. Average and standard deviation (SD) of impedimetric data are also reported in the supplementary document in the format of box and whisker plots. 

We considered four other cut-offs using this estimation and calculated and compared the receiver operating curve or ROC and AUC for them for the precise selection of classification criteria (Figure 3B). Additionally, it can be inferred from Figure 3B that solitary classification of thyroid nodules with only Z_1kHz_ or IPS was not satisfying at all (0.7 and 0.77 AUC). Results of the comparison show maximum AUC and related sensitivity/specificity for the predefined parameter cut-offs (3000 Ω and 8.5) (Figure 3C). 

The AUC, sensitivity, and specificity of some patients’ clinical indications, such as age, sex, nodule size, TI-RADS, and Bethesda, have been compared with the TN-IMS score in all tested samples (including thyroid nodules and normal thyroid tissues). As illustrated in the figure, the best classification outcome was obtained by TN-IMS scoring with AUC = 0.89, *p* < 0.001, 92% sensitivity, and 85% specificity (Figure 3D). Also, another comparison was performed between clinical indications and TN-IMS score in only the thyroid nodules (Figure 3E). Although Bethesda scoring has an acceptable relation with permanent pathology scores with *p* < 0.001 and AUC = 0.82, it suffers from an unacceptable combination of sensitivity/specificity (74% and 85%). This occurs when TN-IMS scoring in thyroid nodules leads to 95% sensitivity, 72% specificity, and AUC = 0.83. TI-RADS scoring as the first clinical thyroid nodule evaluation had only 0.71 AUC and unsatisfying sensitivity and specificity of 68% and 65%. 

Table 2 provides an overview of TN-IMS scoring value in several situations, such as intraoperative diagnosis (besides frozen-section pathology), Bethesda II, or Bethesda III and Bethesda IV nodules as intermediate thyroid nodules. Additionally, evaluation of TN-IMS in micro-PTC, TI-RADS3 nodules, and nodules with permanent pathology of follicular variant of PTC is shown in Table 2.

Eight out of eight nodules with intraoperative frozen-section pathology were diagnosed precisely with TN-IMS, according to Table 2. Two PTCs, three micro-PTCs, and one HCC were positive by both frozen section pathology and TN-IMS scoring. 

All the nodules in Bethesda III and Bethesda IV categories (13 nodules) were truly diagnosed with TN-IMS. Additionally, all the malignant nodules with TI-RADS3 scoring were positively scored by TN-IMS. 

Notably, there were 18 nodules with micro-PTC permanent pathology results in the cohort study, in which 12/18 were previously detected in sonographic evaluations. Five of eighteen were not detected in radiological evaluations but incidentally found in the surgical assessment. One nodule was only detectable in permanent pathological examinations.

**Figure 4:**
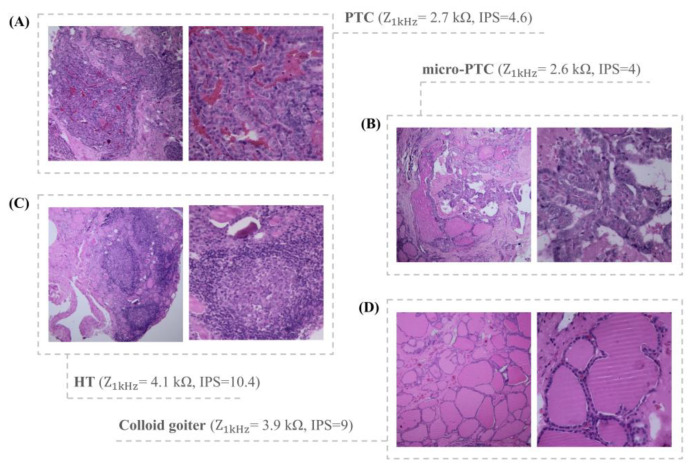
Picture of H&E assays of nodules correctly diagnosed with TN-IMS. (**A**) PTC. (**B**) Micro-PTC. (**C**) HT. (**D**) Colloid goiter.

It is also remarkable that TN-IMS diagnosed all follicular variants of PTC. Indeed, some diagnosed types of thyroid nodules with TN-IMS are illustrated in Figure 4.

## 4. Discussion

The obtained results confirm the sensitivity of TN-IMS in diagnosing high-risk thyroid nodules, which is more valuable when we focus on the benefits of utilizing such a system in surgery. 

Considering permanent pathology as the gold standard, TN-IMS performed more accurately than frozen-section pathology. For example, TN-IMS positively diagnosed a TI-RADS3/Bethesda III nodule (ID #28) with intraoperative frozen pathology result of Hurthle cell neoplasm and Hurthle cell carcinoma. Another fault of frozen-section pathology was in a patient with a micro-PTC nodule underdiagnosed by frozen-section to Hashimoto thyroiditis (patient ID #89, TI-RADS4, Bethesda III). The so-called nodule was reported as micro-PTC in permanent pathology.

In five benign follicular nodule FNA diagnoses (Bethesda II), two nodules with positive TN-IMS scores were diagnosed with PTC in permanent pathology (ID #40 and 101). Two nodules with negative TN-IMS scores were also diagnosed negative in permanent pathology (one HT, ID #52, and one MNG, ID #41), and the other was mistakenly diagnosed as positive by TN-IMS (MNG, ID #51). Therefore, TN-IMS correctly diagnosed 4/5 of the samples that had been declared as benign follicular nodules while FNA missed two PTC nodules.

In the Bethesda III category (nodules with AUS/FLUS cytology results), TN-IMS accurately diagnosed all nine malignant and high-risk masses in accordance with permanent pathology.

Also, four thyroid follicular neoplasms or nodules that were suspicious for follicular neoplasm in cytopathology (Bethesda IV) were finally diagnosed as papillary carcinoma (two PTCs and two micro-PTCs) in permanent pathology. TN-IMS scored all these Bethesda IV nodules as positive or high-risk for malignancy which were in accordance with permanent pathology. Therefore, TN-IMS can be very advantageous in diagnosing follicular neoplasms due to the low sensitivity of frozen-section pathology in these nodules [30]. There were also four Hurthle cell lesions in the cohort study. Three nodules were finally diagnosed with Hurthle cell carcinoma in permanent pathology, and TN-IMS also diagnosed all of them as positive for malignancy (ID #10, 11, 28). Only one of these three nodules (ID #28) was examined with an intraoperative frozen-section pathology. This nodule diagnosed Hurthle cell neoplasm in frozen-section pathology, which is not a definite diagnosis for surgical management. Hurthle cell carcinoma was finally confirmed in the permanent pathology. The other nodule (ID #17) was diagnosed as Hurthle cell neoplasm in frozen-section pathology but Hurthle cell adenoma in permanent pathology. TN-IMS positively scored this nodule. However, Hurthle cell adenoma is not in the malignant category but is an excision-required nodule that should undergo lobectomy [31] or even thyroidectomy [32]. Therefore, we considered this a true positive diagnosis for TN-IMS.

It was impressive that all micro-PTCs challenging in radiological and cytological evaluations were diagnosed positive with TN-IMS. Therefore, TN-IMS can diagnose small suspicious nodules, especially in multinodular patients. Also, the cohort study included 11 follicular variants of PTC as the final pathology was thoroughly diagnosed as true positive by TN-IMS. 

So, an overall review of the obtained results confirms the ability of TN-IMS to detect the benign or malignant nature of follicular or Hurthle cell neoplasms (Table 2). 

Moreover, according to permanent pathological evaluations, Hashimoto thyroiditis was diagnosed in only three patients in the cohort study. Two nodules (ID #73, 91) had AUS results (Bethesda III) in cytopathology; the other (ID #52) was reported as a benign follicular nodule (Bethesda II). All these nodules were scored negative by TN-IMS intraoperatively.

Finally, regarding the high probability of malignancy in TI-RADS3 nodules in the cohort study (Table 2), comparing the TN-IMS results as a radiology assistant under US guidance may be our subsequent research interest.

## 5. Conclusions

We designed a new platform based on an impedance sensing procedure to distinguish high-risk thyroid nodules intra-operatively before starting any dissection. The system named TN-IMS can detect these nodules by a single G20 needle with sensitivity and specificity of 92%, and 85%, respectively, tested on 103 samples. High-risk nodules meaningfully showed different Z_1kHz_ and IPS. TN-IMS may be helpful for surgeons to score newly found nodules or multinodular lesions without any presurgical radiologic reports. Additionally, it may facilitate decision-making when undertaking a lobectomy or total thyroidectomy, especially in the absence of a frozen section pathology.

## Figures and Tables

**Table 1:** Basic information of patients included in the study cohort.

Characteristic	Category	Abundance
Age (years)	>40	31 (56%)
	≤40	24 (44%)
Sex	Female	47 (85%)
	Male	8 (15%)
Nodule Size	<1 cm	28 (36%)
	Between 1 and 3 cm	34 (44%)
	>3 cm	16 (20%)
TI-RADS	2	2 (2%)
	3	20 (19.3%)
	4	25 (24.3%)
	5	24 (23%)
	Not detected	7 (7%)
	No nodule	25 (24.3%)
Bethesda	I, II, III	18 (17.5%)
	IV	4 (3.8%)
	V	21 (20.4%)
	VI	21 (20.4%)
	No FNA reports	14 (13.6%)
	Normal thyroid tissue	25 (24.3%)
Pathological Type	PTC	42 (40.7%)
	Micro-PTC	18 (17.5%)
	HCC	3 (3%)
	HCN	1 (0.9%)
	HT	3 (3%)
	MNG	11 (10.7%)
	Normal tissue	25 (24.3%)

**Table 2:** TN-IMS capability and other pre-, post-, or intra-surgical thyroid nodule evaluations.

	Permanent Pathological Evaluation			TN-IMS Score			
				TP	TN	FP	FN
Nodules with intraoperative frozen pathology	Positive	PTC	2	3 (100%)	0	0	0
		micro-PTC	3	3 (100%)	0	0	0
		HCC	2	2 (100%)	0	0	0
	Negative	HT	1	0	1 (100%)	0	0
Bethesda II (Benign Follicular nodules)	Positive	PTC	2	2 (100%)	0	0	0
	Negative	MNG	2	0	1 (50%)	1 (50%)	0
		HT	1	0	1 (100%)	0	0
Bethesda III (AUS/FLUS)	Positive	HCN	1	1 (100%)	0	0	0
		HCC	2	2 (100%)	0	0	0
		PTC	2	2 (100%)	0	0	0
		micro-PTC	1	1 (100%)	0	0	0
	Negative	MNG	1	0	1 (100%)	0	0
		HT	2	0	2 (100%)	0	0
Bethesda IV (Suspicious for follicular neoplasm)	Positive	PTC	2	2 (100%)	0	0	0
		micro-PTC	2	2 (100%)	0	0	0
Nodules with micro-PTC post-surgical evaluations	Detected as a nodule in radiological evaluations		12	12 (100%)	0	0	0
	Detected as a nodule in surgical evaluations		5	5 (100%)	0	0	0
	Only detected in permanent pathology		1	1 (100%)	0	0	0
Nodules with TI-RADS3 score in presurgical evaluations	Positive	PTC	9	9 (100%)	0	0	0
		micro-PTC	2	2 (100%)	0	0	0
		HCC	2	2 (100%)	0	0	0
	Negative	-	7	0	5 (71%)	2 (29%)	0
Follicular variant of PTC in post-surgical evaluations			11	11 (100%)	0	0	0

## Data Availability

Some or all datasets generated or analyzed during the current study are not publicly available but are available from the corresponding author at a reasonable request.

## Supplementary Materials

The following supporting information can be downloaded at: https://www.mdpi.com/article/10.3390/diagnostics12122950/s1.
