# Supplementary material for: Intraoperative Assessment of High-Risk Thyroid Nodules Based on Electrical Impedance Measurements: A Feasibility Study
Source: Diagnostics (Basel). 2022 Nov 25;12(12):2950. doi: 10.3390/diagnostics12122950 (PMC9776834; doi:10.3390/diagnostics12122950)
Supplement: Supplementary file 1 [file diagnostics-12-02950-s001.zip › diagnostics-1956213-supplementary.pdf]

Supplementry

- **Multiphysics simulation**

A multiphysics simulation was performed to better understand electrical current penetration depth into the thyroid gland, find the most sensitive area of the sensor, and investigate different dimensional parameters on the TN-IMS response, such as ECG chest lead electrode surface area, electrode distances, *etc*. A similar geometry of the neck structure was designed comprising a thyroid gland with a cancerous thyroid nodule and peripheral strap muscles(**Supplementary Figure S1**). The conductivity and relative permittivity of each part are used in multiphysics equations. All the electrical parameters, such as constant voltage amplitude and sweeping frequency range, are adjusted according to the actual situations in the test.

As illustrated in **Supplementary Figure S1**A, the maximum electrical current density in the whole geometry belongs to the needle tip with J= 543 A/m^2 and next to the periphery of the ECG chest lead with J= 90 A/m^2 in f=500 kHz. So, the electrical current density at the needle tips is six times greater than the ECG chest lead area, and the most sensitive area in the TN-IMS system is the needle tip. Also, **Supplementary Figure S1**B shows that electrical current density increases remarkably by increasing the frequency from 1Hz to 1MHz. In contrast, changing the ECG chest lead diameter or distance between the two electrical contacts slightly changes the electrical current density(C and D). This analysis ensured us that some undesirable changes in the distance of two electrodes due to anatomical differences in different patients have no remarkable effect on the TN-IMS measured response. Also, the electrical current penetration depth of the needle into the tissue is about 1mm(**Supplementary Figure S1**E). It is measured by changing the tumorsphere lateral distance from the needle tip in some steps and measuring the critical point in which a drastic

change of about 98% has occurred in the electrical current density. This result shows that the needle should be completely inserted into the suspicious nodule for accurate measurement.

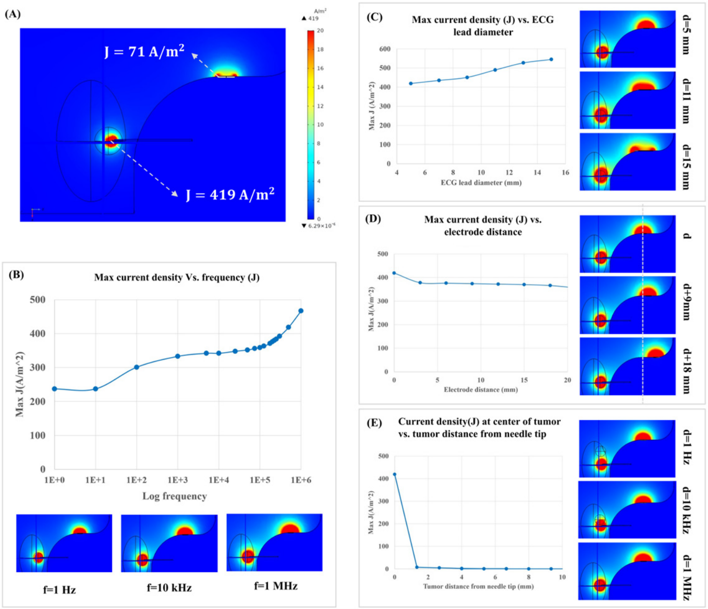

Supplementary Figure S1: Electrical simulation of TN-IMS. (A) Representation of max electrical current density at electrode places. The needle tip is the most sensitive electrode of the system. (B) Maximum current density(i.e., the current density at the needle tip) versus frequency changes from 1 Hz to 1 MHz. Changing the frequency leads to a drastic increase in max current density. (C) Effect of changing ECG chest lead diameter in the maximum electrical current density at f=500kHz. The current density increases slightly by increasing the ECG chest lead diameter. (D) Effect of increasing the distance of two electrodes in the maximum electrical current density at f=500kHz. The electrode distance does not have a considerable impact on the responses. (E) Simulation of electrical current penetration depth into the thyroid nodule. The transition point is about 1 mm distance from the needle tip.

Measuring electrical current flowing through the human body is mandatory for the safety evaluation of a medical device. The measurement device of TN-IMS uses a 3.6V rechargeable battery power that only can be used when it is not connected to domestic electricity via the electric charger. So, it is completely isolated from the main electricity source. Also, we designed a setup to measure the alternating electrical current through a sample tissue on a rabbit model. The ECG

chest lead was connected to the rabbit's flank, and the needle was inserted into its leg muscle. The distance between the two electrical connectors was adjusted to be about the same distance between the submental skin and thyroid gland (about 5cm). The measurement was accomplished, and a minimum impedance magnitude of 2500 ohm was recorded for the whole frequency sweep range. As the applied constant voltage amplitude is 0.4V, the alternating electrical current amplitude is 160μA(<<1mA) and in the safe margin of electrical measurements without any electrical shock to the body. The transmitted electrical current is also in the low range of about 0.64mW.

- ## Mean and Standard Deviation(SD) of classification parameters

As described in the manuscript, the TN-IMS diagnosis is based on two classification parameters of Z_{1kHz}-IPS. So, separate statistics such as average and standard deviation can not reflect the classification performance. To overcome this issue, we considered a new parameter by multiplying Z_{1kHz} and IPS (Z_{1kHz}×IPS) and calculated the mean and SD in the box and whisker plot in **Supplementary Figure S2**.

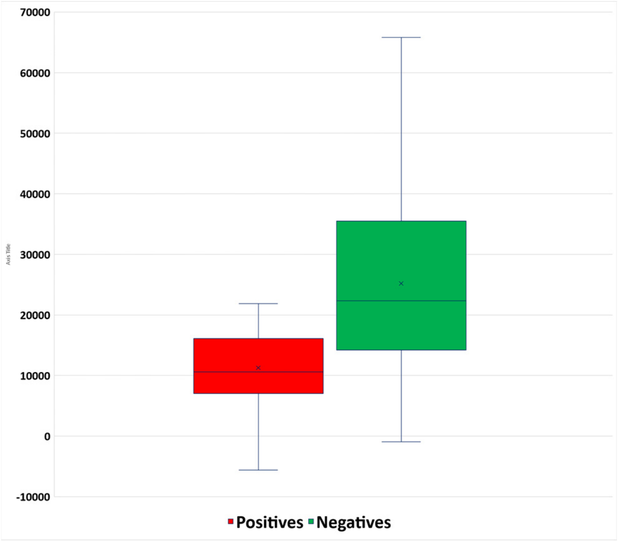

Supplementary Figure S2: Mean and SD for Z_{1kHz}×IPS

Although the distinction between benign and malignant thyroid nodules is performed more effectively than separate analyses of Z_{1kHz} and IPS, it does not reflect the classification accuracy. It is because of the heterogenous structure that a thyroid nodule may have. For example, a PTC in the background of Hashimoto thyroiditis has a lower Z_{1kHz} than a PTC in the background of a colloid goiter. So, the IPS is used to differentiate these two categories. It means that the co-consideration of Z_{1KHz} and IPS resulted in our cut-off calibration.
